# Time-Lapse Imaging Reveals Symmetric Neurogenic Cell Division of GFAP-Expressing Progenitors for Expansion of Postnatal Dentate Granule Neurons

**DOI:** 10.1371/journal.pone.0025303

**Published:** 2011-09-23

**Authors:** Takashi Namba, Hideki Mochizuki, Ryusuke Suzuki, Masafumi Onodera, Masahiro Yamaguchi, Hideo Namiki, Seiji Shioda, Tatsunori Seki

**Affiliations:** 1 Department of Anatomy, Juntendo University School of Medicine, Tokyo, Japan; 2 Integrative Bioscience and Biomedical Engineering, School of Science and Engineering, Waseda University, Tokyo, Japan; 3 Department of Neurology, Kitasato University School of Medicine, Kanagawa, Japan; 4 Department of Anatomy, Showa University School of Medicine, Tokyo, Japan; 5 Department of Hematology, Institute of Clinical Medicine, University of Tsukuba, Ibaraki, Japan; 6 Departments of Otolaryngology and Physiology, Graduate School of Medicine, University of Tokyo, Tokyo, Japan; 7 Department of Histology and Neuroanatomy, Tokyo Medical University, Tokyo, Japan; University of South Florida, United States of America

## Abstract

Granule cells in the hippocampus, a region critical for memory and learning, are generated mainly during the early postnatal period but neurogenesis continues in adulthood. Postnatal neuronal production is carried out by primary progenitors that express glial fibrillary acidic protein (GFAP) and they are assumed to function as stem cells. A central question regarding postnatal dentate neurogenesis is how astrocyte-like progenitors produce neurons. To reveal cell division patterns and the process of neuronal differentiation of astrocyte-like neural progenitors, we performed time-lapse imaging in cultured hippocampal slices from early postnatal transgenic mice with mouse GFAP promoter-controlled enhanced green fluorescent protein (mGFAP-eGFP Tg mice) in combination with a retrovirus carrying a red fluorescent protein gene. Our results showed that the majority of GFAP-eGFP+ progenitor cells that express GFAP, Sox2 and nestin divided symmetrically to produce pairs of GFAP+ cells (45%) or pairs of neuron-committed cells (45%), whereas a minority divided asymmetrically to generate GFAP+ cells and neuron-committed cells (10%). The present results suggest that a substantial number of GFAP-expressing progenitors functions as transient amplifying progenitors, at least in an early postnatal dentate gyrus, although a small population appears to be stem cell-like progenitors. From the present data, we discuss possible cell division patterns of adult GFAP+ progenitors.

## Introduction

The granule cells of the hippocampal dentate gyrus are produced mainly during the early postnatal period, and neurogenesis continues throughout life [1], [2], [3], [4]. The neurogenic activity is implicated in physiological conditions, such as learning, enriched environments and stress, and also pathological conditions such as temporal epilepsy, ischemia and mental diseases [4], [5], [6], [7], [8], [9]. Understanding these physiological and pathological regulatory mechanisms of postnatal neurogenesis requires detailed knowledge of the neurogenic processes of neural progenitor cells.

Interestingly, the persistent neuronal production from early postnatal to adult stages is carried out by astrocyte-like progenitor cells that express glial fibrillary acidic protein (GFAP) [10], [11], [12]. The course of neurogenesis from astrocyte-like progenitors has been well investigated in the adult hippocampal neurogenic zone and subgranular zone (SGZ), mainly by pulse-chase experiments with BrdU. The primary progenitors (Type 1 or B cells) have astrocytic features that include expression of GFAP in addition to radial morphology and nestin expression [2], [10], [11], [12], [13], [14], [15], [16], [17]. The primary progenitors are thought to divide slowly and generate the subsequent intermediate progenitor and another primary progenitor. The next intermediate or amplifying progenitor (Type 2–3, or D cells) expressing neuronal markers such as Hu, Neurogenin2, Tbr2, PSA-NCAM and DCX is considered to divide rapidly to produce immature neurons or neuron-committed progenitors [13], [18], [19], [20].

GFAP-expressing neurogenic progenitors are also found in the early postnatal dentate gyrus [2], [21], although the early postnatal dentate gyrus has a broader neurogenic region which corresponds to nearly the entire hilus and subgranular zone (SGZ) [1], [2]. In the early postnatal neurogenic zones, a majority of proliferating cells are astrocyte-like cells expressing GFAP, GLAST, nestin and S100β, most of which are not typical radial cells, but are round or elongated cells with relatively short processes and which finally differentiate into granule cells [2], [21]. A previous study using GFAP-Cre mice demonstrates the origin of postnatally generated neurons to be the GFAP+ progenitor [10]. During the early postnatal period, astrocyte-like proliferating cells fill the entire areas of the early postnatal neurogenic zones, hilus and SGZ transiently, but with aging the neurogenic zones gradually become restricted to the SGZ [1], [2].

Despite these extensive studies, there is no information as to the actual cell division patterns of GFAP+ primary progenitors, which is essential to determine the exact profile of progenitor cells. In the developing neocortex, precise knowledge about the property of progenitors has been acquired by observation of the cell division pattern using a time-lapse imaging system [22], [23], [24]. In the present study, to reveal the dynamic cell division patterns and neuronal differentiation processes of GFAP+ primary progenitors, we performed time-lapse imaging analysis of hippocampal slices from postnatal days (P) 4–6 in transgenic mice with mouse GFAP promoter-controlled enhanced green fluorescent protein (mGFAP-eGFP Tg mice) [25]. We used postnatal hippocampal slices in the present study for the following reasons: 1) adult slices are generally not suitable for organotypic slice cultures [26], 2) even in the early postnatal period, dentate granule neurons are produced by GFAP+ progenitors [2], and 3) in slice cultures of the early postnatal hippocampus, GFAP progenitors can differentiate into neurons [21], [27]. In the present time-lapse imaging analysis, we employed a short-term slice culture system using collagen-coated glass bottom dishes that we developed previously [20], because this system provides sharper images than the commonly used filter culture systems which are typically employed for hippocampal organotypic cultures [28], [29], [30], and long-term culture in filter culture systems results in a significant reduction of the capacity of neurogenic activity of proliferating progenitor cells [21]. The present time-lapse experiments in this slice culture system revealed that a major symmetric cell division pattern of astrocyte-like progenitors gave rise to neurons through progenitors expressing GFAP and neuronal markers, simultaneously.

## Results

### Characterization of dividing cells in the neonatal dentate gyrus

**Figure 1:**
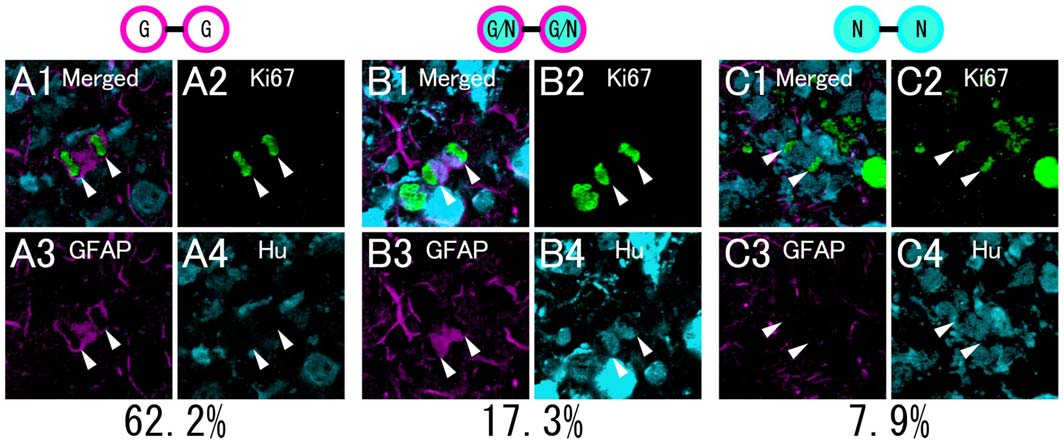
Characterization of dividing cells in the neonatal dentate gyrus. P5 mice were fixed and then processed for immunohistochemistry. The prepared slices were stained with Ki67 (A2, B2, C2), GFAP (A3, B3, C3) and Hu (A4, B4, C4). A: A Ki67+ dividing cell pair expresses GFAP but not Hu. B: A Ki67+ dividing cell pair expresses both GFAP and Hu. C: A Ki67+ dividing cell pair expresses Hu but not GFAP. Scale bar, 10 µm.

To analyze the nature of dividing cells in the early postnatal dentate gyrus, dividing cell pairs in late anaphase and telophase stained with antibodies to Ki67, GFAP and Hu were counted in the hilus and SGZ. All dividing cell pairs symmetrically expressed molecular markers, of which 62.2% were GFAP+, 17.3% were GFAP+/Hu+ and 7.9% were Hu+ (Fig. 1). This suggests that most dividing cells in the early postnatal dentate gyrus expressed GFAP, in agreement with our previous BrdU-labeling experiments, which also demonstrated that GFAP+ proliferating cells are able to differentiate into neurons *in vivo* and *in vitro* [2], [21].

### Properties and division patterns of eGFP+ proliferating cells

**Figure 2:**
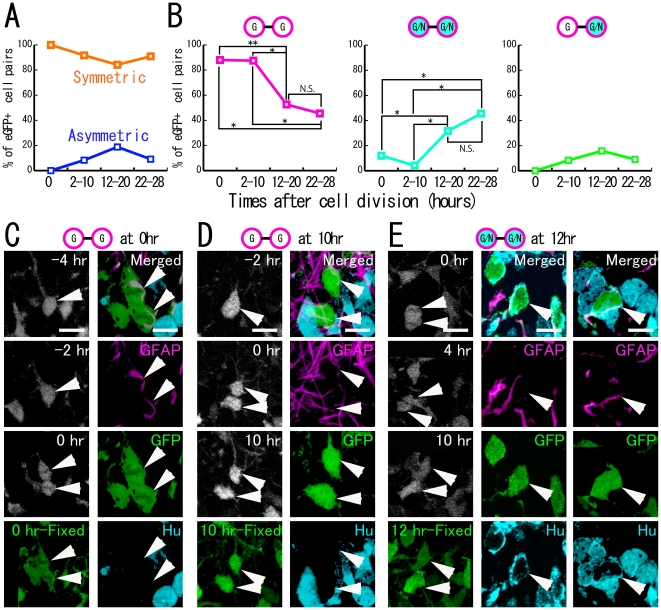
Fates of eGFP+ daughter cells. A: The percentage of eGFP+ cell pairs expressing cell-type specific markers symmetrically or asymmetrically is shown according to the time after cell division. B: The percentage of eGFP+ cell pairs expressing GFAP or Hu is shown according to the time after cell division. C, D, E: Time-lapse imaging of eGFP+ cells (left column) and daughter cell fates at the end of culture (others). E: In the 12-hour fixed section, optical images were projected. Since each cell (arrowheads) was located at a different level of the Z-axis, they are shown separately in the middle and right columns as single z-plane images. Full time-scale images and videos are shown in the supplementary information. G: GFAP+ cells, G/N: GFAP+/Hu+ cells. Scale bar, 10 µm.

To analyze the fate of GFAP+ dividing cells, we performed time-lapse imaging of cultured hippocampal slices obtained from GFAP-eGFP Tg mice. In this experiment, we focused on dividing eGFP-positive (eGFP+) cells in the SGZ and hilus of the dentate gyrus. In total, the fates of 79 daughter cell pairs were monitored. After culture, the hippocampal slices were fixed, and the fates of the daughter cells were examined by immunohistochemistry. The time at which 1 eGFP+ cell divided into 2 daughter cells was regarded as time zero (0 hours) (Figure S1). The properties of the daughter cells at the end of the culture were analyzed at 4 culture times after cell division, namely, 0 hours (n = 25 pairs), 2–10 hours (n = 24 pairs), 12–20 hours (n = 19 pairs) and 22–28 hours (n = 11 pairs) (Fig. 2A), because the cell cycle durations of postnatal dentate progenitors are estimated to be 12–14 hours [31] or 24.7 hours [32].

At 0 hours, the majority of eGFP+ daughter cell pairs expressed GFAP (88.0%, Fig. 2B) and the progenitor cell marker nestin (Figure S2). Only 12.0% of the cell pairs were positive for both GFAP and Hu. Neither the Hu+ cell pair nor the asymmetric pair was observed at 0 hours. At each time window, the proportion of the number of 2 identical daughter cell pairs among all eGFP+ daughter cell pairs was much higher than that of 2 different daughter cell pairs (0 hours, 100% vs. 0% [25 pairs vs. 0 pairs]; 2–10 hours, 91.7% vs. 8.3% [22 pairs vs. 2 pairs]; 12–20 hours, 84.2% vs. 15.8% [16 pairs vs. 3 pairs]; 22–28 hours, 90.9% vs. 9.1% [10 pairs vs. 1 pair]), suggesting that the principal division mode of GFAP-expressing progenitor cells was symmetric (Fig. 2A).

### Fates of symmetrically divided eGFP+ cells

The percentages of the GFAP+ cell pairs and GFAP+/Hu+ cell pairs changed during the culture period. Specifically, the percentage of GFAP+ cell pairs decreased from 88.0% at 0 hours to 45.5% at the end of culture, whereas the percentage of GFAP+/Hu+ daughter cell pairs increased from 12.0% to 45.5% with time after cell division (Fig. 2B, C, D, E; Figs. S2, S3, S4; Videos S1 and S2). This reciprocal change suggests that GFAP+ daughter cells gradually differentiated into GFAP+/Hu+ cells after cell division. To verify the neuronal identity of the GFAP+/Hu+ cells, we attempted to characterize these cells using three different marker antibodies for the neuronal lineage-committed cells, namely, Tbr2, NeuroD and Prox1. These markers can distinguish the different stages of the neuronal lineage-committed cells (Tbr2: early neuronal progenitors; NeuroD: late neuronal progenitors; Prox1: granule cells) [33], [34]. In the P5 GFAP-eGFP mouse hippocampus, the eGFP+/GFAP+/Hu+ cells were rarely labeled by NeuroD and Prox1 antibodies. In contrast, 91.1% of the eGFP+/GFAP+/Hu+ cells expressed Tbr2 (a total of 135 cells were counted; Figure S5), suggesting that the GFAP+/Hu+ daughter cells are early-stage neuron-committed cells. Although the GFAP+/Hu+ cells may have finally differentiated into immature neurons, eGFP cells solely expressing neuronal markers, such as Hu or NeuroD, were not detected in the present time-lapse observation, possibly because neuronal differentiation was accompanied by a decrease in GFAP promoter activity resulting in a loss of eGFP.

**Figure 3:**
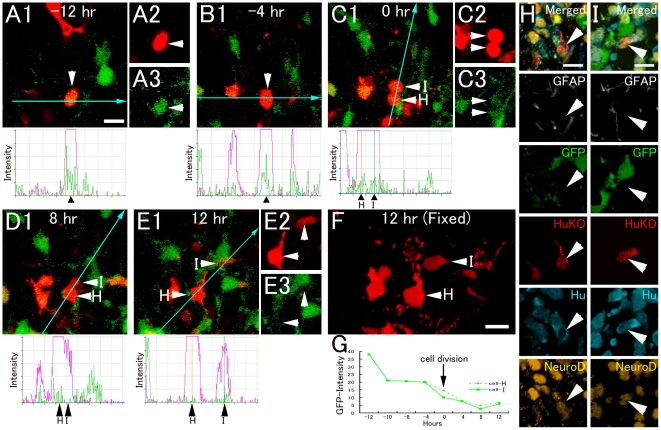
Symmetrical neuronal fate of GFAP+ progenitor cells. A–E, G: Time-lapse imaging of GFP+/HuKO+ cells. The eGFP signals (arrows) gradually decreased, and finally reached background level (A–E, G: fluorescent signal intensity chart). F: Optical images. H–I: Since each cell (arrowheads) in F was located at a different level of the Z-axis, the cells are shown separately in H and I. At the end of culture, both HuKO+ daughter cells expressed Hu and NeuroD. Scale bar, 10 µm.

As 40.9% of the daughter cells lost eGFP fluorescence during the observation period, we could not determine the final fates of these cells. To determine if they finally differentiated into neurons, a retrovirus carrying a red fluorescent protein (HuKO) was injected into the dentate gyrus of P3–5 mGFAP-eGFP Tg mice to label the dividing eGFP+ cells. We monitored 180 eGFP+/HuKO+ cells and found 14 HuKO+ cells that lost eGFP expression during the observation period. At the end of the culture, all cells were positive for Hu and NeuroD, but negative for GFAP (Fig. 3). These results suggest that the majority of GFAP+ progenitors finally differentiated into Hu+/NeuroD+ neurons through a Hu+/GFAP+ transient state (Fig. 3).

**Figure 4:**
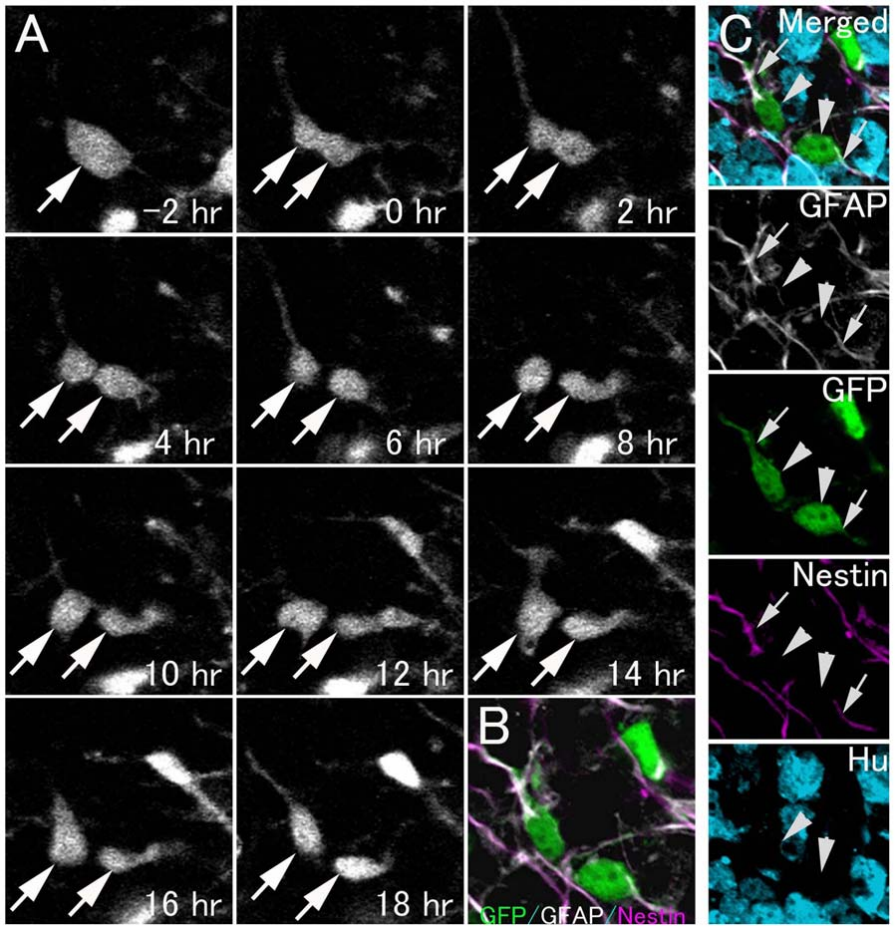
Symmetric division of eGFP+ cells to produce 2 GFAP+/nestin+ cells. A: Time-lapse imaging of GFP+ cell division in a hippocampal slice from a P6 GFAP-eGFP Tg mouse. B: 18 hours after cell division, the slice was fixed and then processed for immunohistochemistry. Optical images C: Both eGFP+ daughter cells (arrowheads) had a radial process and expressed an astrocytic cell marker (GFAP) and a progenitor cell marker (nestin) (arrows), suggesting the self-renewal of a progenitor cell.

Although the percentage of 2 GFAP+ cell pairs decreased as the culture time after cell division increased, half of the symmetrically divided eGFP+ cells were still positive for GFAP. These GFAP+ daughter cell pairs appear to be primary progenitor cells (self-renewing) and astrocytes (astrogliogenesis). As most of the GFAP+ symmetrically divided daughter cell pairs were also positive for nestin, a marker for progenitor cells (83.3%, Fig. 4). In addition, most eGFP+/GFAP+/Hu− cells also expressed another progenitor cell marker, Sox2 [34], [35] in the P5 GFAP-eGFP mouse hippocampus (99.1% of a total of 113 cells; Figure S6) and both of the GFAP+/Hu− symmetrically divided daughter cells expressed Sox2 in the cultured slices (Figure S7), suggesting that the GFAP+/Hu− daughter cells may possess the properties of neurogenic primary progenitors. This is also supported by our previous studies showing that approximately 70% to 80% of progenitor cells of the early postnatal dentate gyrus become neurons *in vivo* [2]. To clarify whether or not the GFAP+ daughter cells are neurogenic progenitors, longer range time-lapse imaging is needed.

### Fates of asymmetrically divided eGFP cells

**Figure 5:**
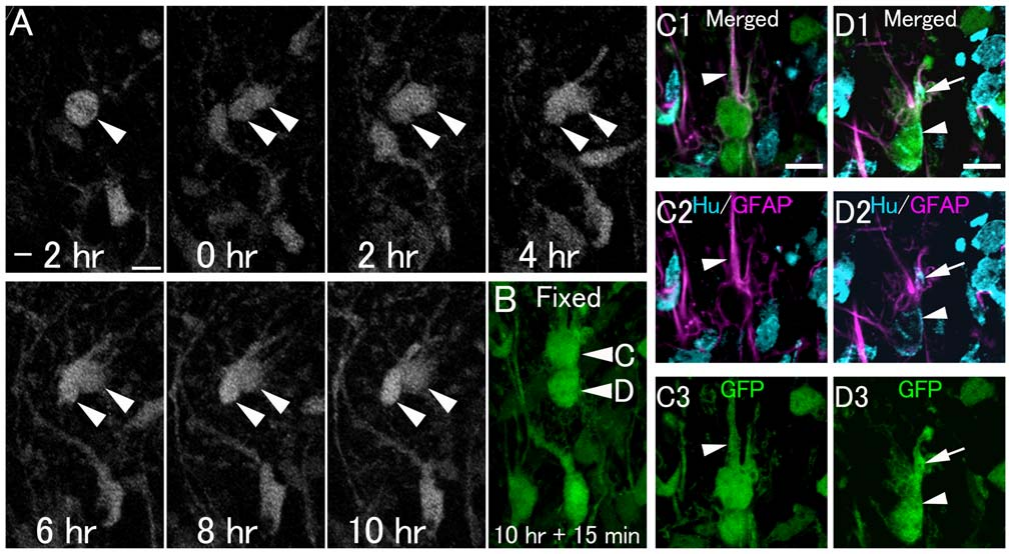
Asymmetric division of eGFP+ cells to produce a GFAP+ radial type cell and a neuronal cell. A: Time-lapse imaging of GFP+ cell division in a hippocampal slice from a P6 GFAP-eGFP Tg mouse. Also see Video S3. B: 10 hours after cell division, the slice was fixed and then processed for immunohistochemistry. Optical images (C, D): The cells in B indicated by arrowheads C and D are located at different levels of the Z-axis. Each cell is shown separately in different single optical images of C and D. The GFP-positive cells (arrowheads C and D) in B correspond to those indicated by arrowheads in C and D, respectively. One GFP+ daughter cell has a radial process and an astrocytic cell marker (GFAP+). Another GFP+ daughter cell expresses GFAP and the neuronal marker Hu, suggesting a neuronal lineage-commitment. Scale bar, 10 µm.

In addition to symmetric cell division, less than 10% of eGFP+ cells divided asymmetrically and produced a GFAP+/Hu+ cell and a GFAP+ cell (Fig. 2A, Fig. 5, Video S3). This suggests the possibility that GFAP+ progenitor cells produced a cell pair consisting of a neuron-committed cell and a self-renewed GFAP+ cell by asymmetric cell division. However, we could not confirm if GFAP+/Hu+ cells finally developed into immature neurons that were positive only for neuronal markers, because of the decrease in eGFP expression in neuron-committed cells.

**Figure 6:**
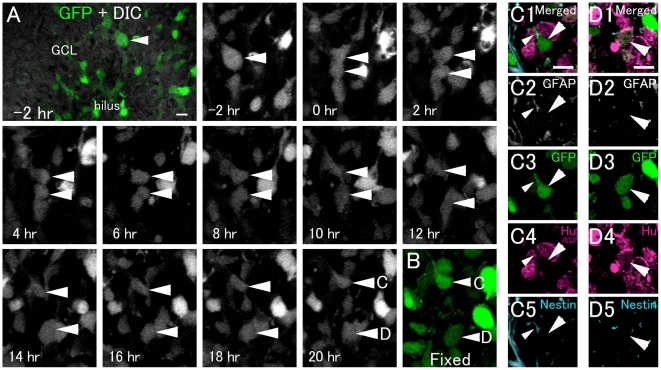
Asymmetric division of GFP+ cells to produce a GFAP+/nestin+ radial type cell and a neuronal cell. A: Time-lapse imaging of GFP+ cell division in a hippocampal slice from a P5 nestin-GFP Tg mouse (see Video S4). B: 20 hours after cell division, the slice was fixed and then processed for immunohistochemistry. Optical images (C, D): The cells in B indicated by arrowheads C and D are located at different levels of the Z-axis. Each cell is shown separately in different single optical images of C and D. The GFP-positive cells (arrowheads C and D) in B correspond to those indicated by arrowheads in C and D, respectively. One GFP+ daughter cell had a radial process and an astrocytic cell marker (GFAP+) and a progenitor cell marker (nestin+). Another GFP+ daughter cell expressed the neuronal marker Hu+, but was negative for GFAP and nestin, suggesting neuronal differentiation. Scale bar, 10 µm.

To examine the neuronal fate of daughter cells produced by asymmetric division of progenitor cells, nestin-GFP transgenic mice were used because nestin-eGFP is reportedly expressed by primary progenitors and the subsequent progenitors express only neuronal markers [13], [36]. Of all nestin-eGFP+ dividing cells, 16.7% of eGFP+ cells divided asymmetrically and produced cell pairs consisting of an Hu+ cell and a GFAP+/nestin+ cell (n = 24 GFP+ cell pairs, Fig. 6, Video S4). These results suggest that asymmetric division of GFAP+ progenitors produced pairs of an immature neuron or neuronal progenitors, and a self-renewed GFAP+/nestin+ primary progenitor.

## Discussion

**Figure 7:**
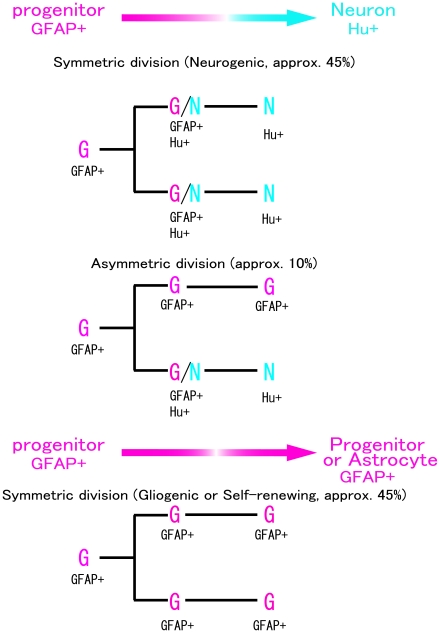
Diagram of cell division patterns of GFAP+ progenitors to produce dentate granule cells during the postnatal period. Most GFAP+ progenitor cells underwent symmetric division to generate neurons through an Hu+/GFAP+ transitional state, or to produce the neurons themselves. A small population of GFAP+ progenitor cells divided asymmetrically and produced both a self-renewed GFAP+ progenitor cell and a neuron.

Using time-lapse analysis with a slice culture of the postnatal hippocampus from mGFAP-eGFP Tg mice and nestin-eGFP Tg mice, we examined how GFP+ proliferating progenitors that express GFAP, Sox2 and nestin divide and differentiate into neuron-committed cells. The present time-lapse observation clearly demonstrated that the neurogenic region in the early postnatal dentate gyrus contained 2 distinct types of GFAP+ “neurogenic” progenitor cells, in addition to GFAP+ progenitors to be able to produce a pair of GFAP+ cells. Judging from the results of immunohistochemistry for GFAP and neuronal markers, the majority underwent symmetric division to produce a pair of neuron-committed cells (45%) or pairs of GFAP+ cells (45%), while a minority of GFAP+ neurogenic progenitors exhibited asymmetric cell division to produce a GFAP+ progenitor cell and a neuron-committed cell (10%; Fig. 7). To date, despite a lack of a time-lapse imaging analysis in postnatal hippocampal neurogenesis, GFAP+ progenitors have been generally considered to be stem cells or stem cell-like progenitors which undergo asymmetric cell division [13], [18], [19], [20], [37]. Unusually, the present time-lapse study has shown that most of the GFAP+ progenitors are not typical stem cells, but progenitors which divide symmetrically to generate a pair of neuronal progenitors or immature neurons, at least in the early postnatal dentate gyrus, although a small fraction of stem cell-like cells dividing asymmetrically also exist. Symmetric cell division in early dentate proliferating cells is supported by a recent paper using retrovirus labeling, although in which the astrocytic properties of the proliferating cells are not identified [38].

Additionally, the present time-lapse study showed that about half of GFP+ dividing cells became Hu+/GFAP+ cells. Most Hu+/GFAP+ cells exhibited Tbr2 expression and their proliferative activity was low, suggesting that the majority of Hu+/GFAP+ cells are on the way to further differentiation into the next neuron-committed cells. Furthermore, retrovirus-HuKO labeling approach revealed that when HuKO-labeled dividing cells lost GFP fluorescence, most of the labeled cells became Hu+/GFAP− neuronal cells. Therefore, the majority of Hu+/Tbr2+/GFAP+ cells appear to be a transient state when GFAP+ astrocytic progenitors differentiate into immature neurons or neuronal precursors, and a minority is likely to be intermediate progenitors that can further proliferate and produce immature neurons. In this regard, our previous immunohistochemical study in the adult hippocampus also indicates the existence of Hu+/GFAP+ proliferating cells [20], [39]. However, the proliferative activity of adult Hu+/GFAP+ cells is relatively high, suggesting a difference in the property of Hu+/GFAP+ progenitors between early postnatal and adult stages, as mentioned later.

### Glial fibrillary acidic protein-positive progenitors function as transient amplifying cells

The cell division pattern and fate of GFAP+ proliferating cells revealed by time-lapse imaging suggest the identity of GFAP+ progenitors in the early dentate neurogenic region. In neocortical development, time-lapse imaging analysis has precisely defined 2 types of progenitors: stem cells and amplifying cells. Stem cells produce single stem cells and single neuron-committed progenitors by asymmetric cell division, or self-renew by symmetric cell division. Transient amplifying cells (also referred to as intermediate progenitors) undergo symmetric cell division to produce a pair of neurons or self-renew [22], [23], [24]. According to the definition of neocortical progenitors, the majority of early postnatal dentate GFAP+ symmetrically dividing progenitors that produced a pair of neuron-committed progenitors or neurons can be identified as transient amplifying cells, while a minority of GFAP+ asymmetrically dividing progenitors appears to be stem cell-like progenitors. The neocortical amplifying cells are considered to play an important role in the increase in the number of neocortical neurons [40], [41], [42]. Similarly, the dentate GFAP+ transient amplifying progenitors would be required for the rapid production of dentate granule cells during the postnatal period [2], [43], [44], [45].

However, we could not clearly define the nature of GFAP+ symmetrically dividing progenitors that produced a pair of GFAP+ cells. There appear to be various possible types of GFAP+ progenitors undergoing symmetrical proliferative division that are either 1) stem cell-like cells that divide during long periods, which self-renew and partially undergo asymmetric cell division; 2) neuron-committed transient amplifying cells that undergo a few or several symmetric divisions, and then produce neurons; or 3) astrocyte-committed transient amplifying cells that symmetrically divide a few or several times and subsequently generate astrocytes. Because astrocyte-like cells and proliferating cells in the postnatal neurogenic zone exhibit an age-dependent decrease, and a majority of astrocyte-like proliferating cells become neurons [2], [10], a majority of GFAP+ progenitors do not appear to be stem cells or gliogenic transient amplifying progenitors, but rather neuron-committed transient amplifying cells. However, there should be a small number which self-renew as stem cell-like cells and give rise to astrocytes. To accurately distinguish these GFAP+ progenitor cells, it is necessary to perform long-term cultures in time-lapse experiments to pursue the progeny of GFAP+ cells over several cell divisions. However, it may be difficult to perform such experiments, because our previous slice culture experiments suggest that the neurogenic capacity of dividing neural progenitors is reduced as the culture period becomes longer [21]. To maintain their capacity for neuronal differentiation, new hippocampal slice culture techniques in long-term time-lapse experiments are required.

### Similarities and differences between postnatal and adult neurogenesis

A fundamental question is whether GFAP+ transient amplifying progenitors exist in the adult dentate neurogenic zone. In the adult hippocampus, GFAP+ progenitors have been typically classified as stem cells or primary progenitors, designated as Type 1 or B cells [12], [13], [36]. The following intermediate or transient amplifying cells are considered to be devoid of GFAP and express proneuronal proteins (mash1 and neurogenin2) and neuronal markers (Hu, PSA-NCAM, DCX, NeuroD and Tbr2), designated as Type 2–3 or D cells [13], [18], [19], [20]. Additionally, because adult GFAP+ progenitors have been speculated to be slowly dividing stem cells which divide asymmetrically [13], [46], the adult SGZ does not appear to contain GFAP+ amplifying cells. However, it should be noted that the concept was mostly based on studies using BrdU-labeling and immunolabeling with glial and neuronal markers.

Several lines of evidence support the notion that the adult hippocampus also contains GFAP+ symmetrically dividing or amplifying neurogenic progenitors. Firstly, our previous studies in rats and GFAP-eGFP transgenic mice have shown that the adult neurogenic zone has 2 types of GFAP+ progenitors with or without neuronal (and proneuronal) markers such as Hu and Mash-1, in addition to the stem cell marker Sox2. The neuronal marker-positive progenitors have relatively higher proliferative activity and partial non-radial morphology, and do not appear to be stem cells, but rather amplifying progenitors [20], [39]. Secondly, several studies have shown an increase in the number of GFAP+ neural progenitors in normal, enriched, running [47], seizure-induced [48] and N-methyl-D-aspartate receptor antagonist-treated animals [49], [50], suggesting symmetric cell division of GFAP+ progenitors since their number will not increase with only asymmetric cell division. Finally, a time course experiment of BrdU-labeled progenitors demonstrates that the increase in the number of GFAP+ progenitors is transient [47]. After an increase in the number of the GFAP+/BrdU+ progenitors, a gradual decrease begins, while the number of neuronal marker-expressing BrdU+ cells increases. This apparently indicates that a daughter cell pair of GFAP+ progenitors becomes neurons, because the number of the GFAP+/BrdU+ progenitors never decreases by only asymmetric cell division. Taken together, these data strongly suggest that the GFAP+ neural progenitors, owing to their transient increase, principally function as transient amplifying progenitors to produce neurons in the adult hippocampus in addition to their role in neural and glial stem cells, as suggested previously [47].

Although these studies suggest the presence of GFAP+ amplifying progenitors from the early postnatal to adult hippocampus, it should also be noted that there could be some differences in the properties of GFAP+ amplifying cells between the two stages. For example, major GFAP+ proliferating cells were Hu-negative in the early postnatal dentate neurogenic regions, but Hu-positive in the adult ones [2], [20], [39]. A study using the neurosphere assay has shown that progenitor cells from the early postnatal dentate gyrus exhibit self-renewal and multipotentiality, but those from the adult dentate gyrus rarely do [51]. It is thus possible that the early postnatal type of Hu−/GFAP+ amplifying progenitors progressively become adult-type Hu+/GFAP+ amplifying progenitors with aging, and that the alternation in the GFAP+ progenitors is associated with age-dependent decrease in postnatal neurogenesis [2], [52], [53], [54]. Further investigation to reveal the mechanism underlying the regulation of cell division patterns and the age-dependent transition of GFAP+ amplifying progenitors will contribute to regenerative brain therapy, particularly regarding the up-regulation of intrinsic postnatal neurogenesis.

## Materials and Methods

### Animals and retroviral injection

All animal studies were approved by the Institutional Animal Care and Use Committee of Juntendo University, Japan. Before conducting any experiments, mGFAP-eGFP or nestin-GFP [55] transgenic mice were deeply anesthetized on ice. To trace newly generated cells, we used our modified retrovirus vector, GCDNsap-humanized Kusabira Orange (HuKO). Details of the construction and titer of this vector have been described previously [56]. For dual-color time-lapse imaging, a retrovirus vector (0.5 µl) was stereotactically injected into the hilus of P3–5 mGFAP-eGFP mice (posterior = 1 mm from the bregma; lateral = 1 mm; ventral = 1 mm), as described previously [2] and time-lapse imaging was scheduled for 2 days after the injection.

### Slice culture preparation

Mice were deeply anesthetized on ice. Hippocampal slices were prepared by the standard method [21], and then the slices (350 µm in thickness) were transferred onto a collagen-coated glass bottom dish. The culture medium was a mixture of 50% minimum essential medium (Invitrogen, Carlsbad, CA, USA), 25% heat inactivated horse serum (Invitrogen) and 25% Hank's balanced salt solution (Invitrogen) supplemented with penicillin-streptomycin-glutamine (Invitrogen). Glucose was added to reach the final concentration of 6.5 mg/ml.

### Time-lapse confocal imaging

Two to 3 hours after hippocampal slice preparation, time-lapse recording was performed manually using an inverted confocal laser scanning microscope (LSM 510 META; Zeiss, Germany) and minimal laser excitation (typically 1% of an Argon 488 laser) to prevent photodamage and photobleaching. Differential interference contrast images were obtained to confirm the granule cell layer. To monitor cell movements, stacks of images were collected in the z-plane every 2 or 4 hours using a 40× objective. Between these time points, slices were kept in a water-jacketed incubator at 34°C, with 5% CO_2_ and 50% O_2_. After time-lapse imaging, cultured slices were fixed overnight in a 4% paraformaldehyde solution at 4°C. Time-lapse sequences were arranged using Photoshop (Adobe Systems Inc., CA, USA) and QuickTime Pro (Apple, Cupertino, CA, USA).

### Quantification of GFP expression in HuKO-labeled cells

To quantify the expression levels of GFP in HuKO-labeled cells, we randomly chose 180 GFP+/HuKO+ cells. The average fluorescence intensities of GFP were calculated using Image-J software. Microscope settings, such as laser power, pinhole size and detector gain, were kept equivalent during time-lapse imaging.

### Immunofluorescence staining of time-lapse imaged slices

The antibodies, concentrations and vendors used are listed in Table 1. Fixed slices were washed with phosphate-buffered saline (PBS). The primary antibodies were diluted with PBS containing 1% bovine serum albumin (BSA), 0.2% Triton X-100 and 10% normal donkey serum, and the secondary antibodies were diluted with PBS containing 1% BSA, 0.1% Triton X-100 and 1% normal donkey serum. All subsequent incubations were carried out with free-floating sections in 10-ml vials using a rotator. Each of the following steps was followed by PBS washing: the slices were incubated overnight for 3 days with a mixture of primary antibodies diluted in the same solution at 4°C. The sections were then incubated at room temperature for 3 hours with a mixture of secondary antibodies. The slices were further incubated at room temperature for 3 hours with streptavidin-Alexa 405 (Invitrogen) (1∶400). Finally, the specimens were mounted on glass slides. For quintuple staining, Cy5 signals were completely photobleached under LSM 510 META (Zeiss) after quadruple imaging, as described below. The slices were then washed with PBS and incubated overnight for 3 days with goat anti-NeuroD diluted in PBS containing 1% BSA, 0.2% Triton X-100 and 10% normal donkey serum at 4°C. The sections were then incubated at room temperature for 3 hours with donkey anti-goat IgG-Cy5. Finally, the specimens were remounted on glass slides. Samples were viewed through LSM 510 META (Zeiss) with 20× and 63× objectives. The images were corrected for brightness and contrast using the Zeiss LSM Image Browser, Adobe Illustrator 9.0 (Adobe Systems Inc.) and Adobe Photoshop 7.0 (Adobe Systems Inc.)

**Table 1:** Antibodies.

Marker	Species, isotype	Label	Working dilution	Vendor
Primary antibodies				
GFAP	Guinea pig IgG	None	1∶800	Advanced ImmunoChem., CA, USA
GFP	Rat IgG	None	1∶400	Nakalai Tesque, Japan
Hu	Human IgG	None	1∶2000	Gift from Dr. H. J. Okano
Ki67	Mouse IgG	None	1∶100	Novocastra, UK
Nestin	Mouse IgG	None	1∶2000	BD Bioscience, CA, USA
NeuroD	Goat IgG	None	1∶400	Santa Cruz Biotech., CA, USA
Sox2	Rabbit IgG	None	1∶1000	Chemicon, CA, USA
Tbr2	Rabbit IgG	None	1∶2000	Gift from Dr. R. Hevner
Secondary antibodies				
Anti-goat IgG	Donkey IgG	Cy5	1∶200	Jackson, PA, USA
Anti-guinea pig IgG	Donkey IgG	Cy3	1∶200	Jackson
Anti-guinea pig IgG	Donkey IgG	Biotin	1∶200	Jackson
Anti-human IgG	Donkey IgG	Cy5	1∶200	Jackson
Anti-mouse IgG	Donkey IgG	Cy5	1∶200	Jackson
Anti-mouse IgG	Donkey IgG	Biotin	1∶200	Jackson
Anti-rabbit IgG	Donkey IgG	Cy3	1∶200	Jackson
Anti-rat IgG	Donkey IgG	Cy2	1∶200	Jackson

### Immunofluorescence staining of fixed hippocampal tissues

Immunohistochemistry was performed as described previously [2]. Briefly, the frozen brains were coronally sliced into 14-µm sections using a cryostat (CM-3000; Leica, Nussloch, Germany). After washing in PBS, the sections were incubated at 4°C overnight with first antibodies in PBS containing 1% bovine serum albumin (BSA), then incubated at room temperature for 1–2 h with secondary antibodies in PBS containing 1% BSA. For immunostaining with anti-Tbr2 antibody, the sections were boiled in 0.01 M citrate buffer for 15 min and washed in PBS prior to the first antibody incubation.

### Statistical analysis

For comparison of 2 groups, statistical significance was assessed using the chi-square test or Mann-Whitney U test. A total of 79 GFAP-eGFP+ daughter cell pairs were counted.

## Supporting Information

Figure S1 **Schematic illustration of time-lapse imaging analysis.** Images are collected in cultured hippocampal slices every two hours. The time at which one eGFP+ cell divides into two daughter cells is regarded as time zero (0 hours). For example, if an eGFP+ cell divides into two daughter cells 12 hours after culture and the slice is fixed 28 hours, this is depicted as “16 hours after cell division”.
(TIF)

Figure S2 **Quadruple staining of eGFP+ daughter cells at 0 hours after cell division.** eGFP+ cells indicated by arrowheads correspond to the cells in Fig. 1B. Both daughter cells (arrowheads) expressed GFAP and nestin, but not Hu.
(TIF)

Figure S3 **Time-lapse imaging of eGFP+ cells (A) and daughter cell fates at the end of culture (B, C).** A: Full time-scale images of eGFP+ cells represented in Fig. 1C. B, C: Two eGFP+ daughter cells at the end of imaging. Both daughter cells expressed GFAP (magenta), but not Hu (blue).
(TIF)

Figure S4 **Time-lapse imaging of eGFP+ cells (A) and daughter cell fates at the end of culture (B, C).** A: Full time-scale images of eGFP+ cells are represented in Fig. 1D. B, C, D: Two eGFP+ daughter cells at the end of the imaging. Both daughter cells expressed GFAP (magenta) and Hu (blue).
(TIF)

Figure S5 **Phenotypic analysis of eGFP+ cells in the dentate gyrus at P5.** The eGFP+ (green)/GFAP+ (white)/Hu+ (blue) cell indicated by arrow is also positive for Tbr2 (magenta). Scale bar, 5 µm.
(TIF)

Figure S6 **Phenotypic analysis of eGFP+ cells in the dentate gyrus at P5.** The eGFP+ (green)/GFAP+ (white)/Hu− (blue) cells indicated by arrows are also positive for Sox2 (magenta). Scale bar, 10 µm.
(TIF)

Figure S7 **Symmetric division of eGFP+ cells to produce 2 GFAP+/Sox2+ cells.** A, B: Time-lapse imaging of GFP+ cell division in a hippocampal slice from a P4 GFAP-eGFP Tg mouse. C: Both eGFP+ daughter cells (arrowheads) expressed an astrocytic cell marker (GFAP) and a progenitor cell marker (Sox2), suggesting the self-renewal of a progenitor cell. Scale bar, 5 µm.
(TIF)

Video S1 **Time-lapse video of GFAP-eGFP+ cells shown in** **Fig. 2D** **and Figure S3.** Arrowheads indicate the eGFP+ mother cell and daughter cells.
(AVI)

Video S2 **Time-lapse video of GFAP-eGFP+ cells shown in** **Fig. 2E** **and Figure S4.** Arrowheads indicate eGFP+ daughter cells.
(AVI)

Video S3 **Time-lapse video of GFAP-eGFP+ cells shown in** **Fig. 5** **.** Arrowheads indicate the eGFP+ mother cell and daughter cells.
(AVI)

Video S4 **Time-lapse video of nestin-GFP+ cells shown in** **Fig. 6** **.** Arrowheads indicate the GFP+ mother cell and daughter cells.
(AVI)
