# Supplementary figures and images for: Time-Lapse Imaging Reveals Symmetric Neurogenic Cell Division of GFAP-Expressing Progenitors for Expansion of Postnatal Dentate Granule Neurons
Source: PLoS One. 2011 Sep 23;6(9):e25303. doi: 10.1371/journal.pone.0025303 (PMC3179506; doi:10.1371/journal.pone.0025303)

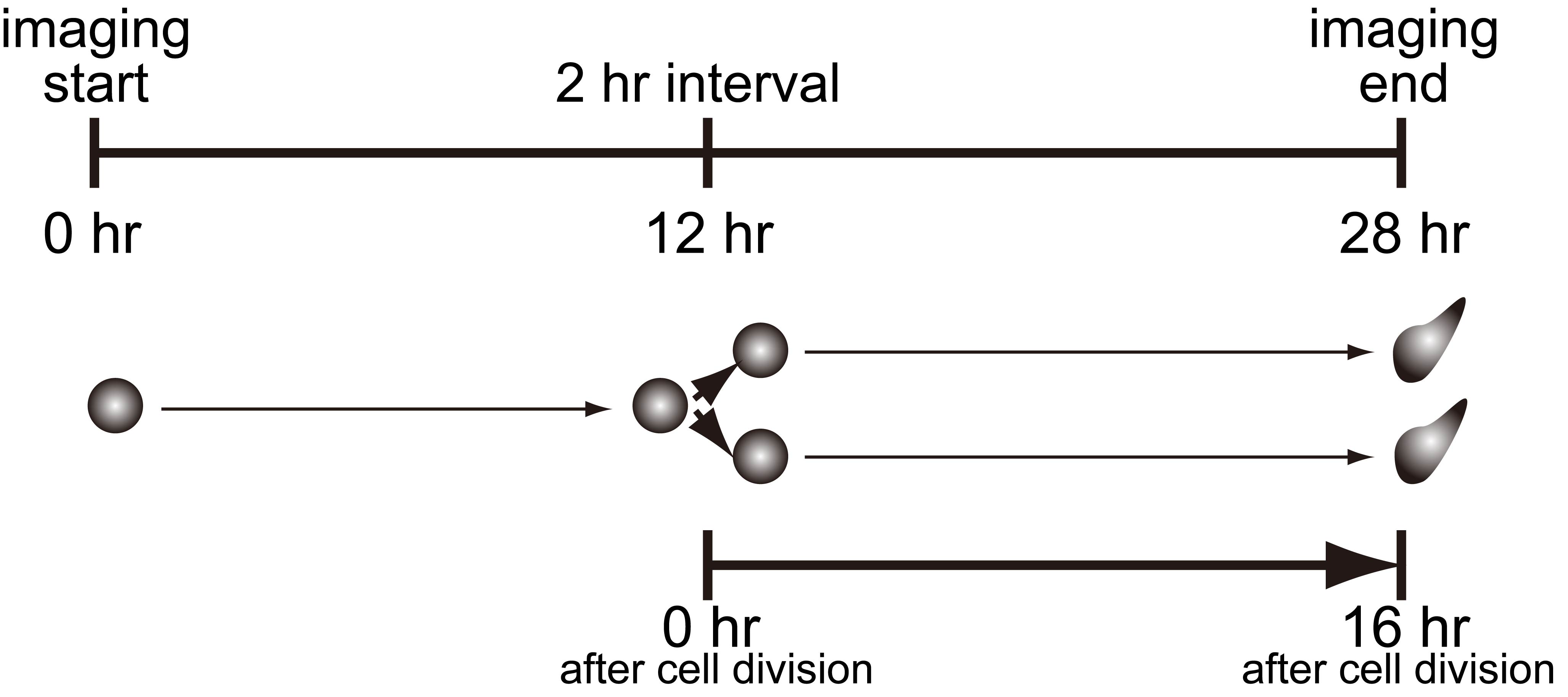

Supplement: Figure S1 — Schematic illustration of time-lapse imaging analysis. Images are collected in cultured hippocampal slices every two hours. The time at which one eGFP+ cell divides into two daughter cells is regarded as time zero (0 hours). For example, if an eGFP+ cell divides into two daughter cells 12 hours after culture and the slice is fixed 28 hours, this is depicted as “16 hours after cell division”. (TIF) [file pone.0025303.s001.tif]

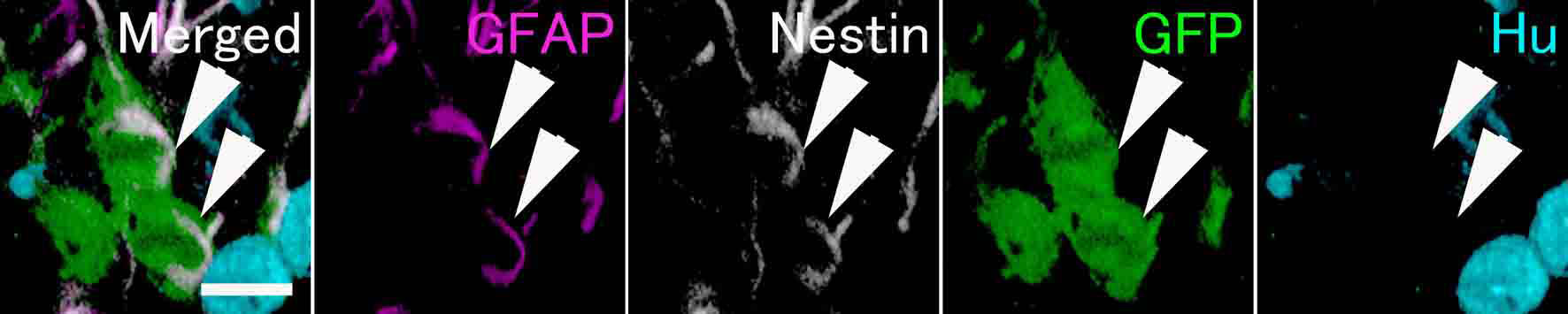

Supplement: Figure S2 — Quadruple staining of eGFP+ daughter cells at 0 hours after cell division. eGFP+ cells indicated by arrowheads correspond to the cells in Fig. 1B. Both daughter cells (arrowheads) expressed GFAP and nestin, but not Hu. (TIF) [file pone.0025303.s002.tif]

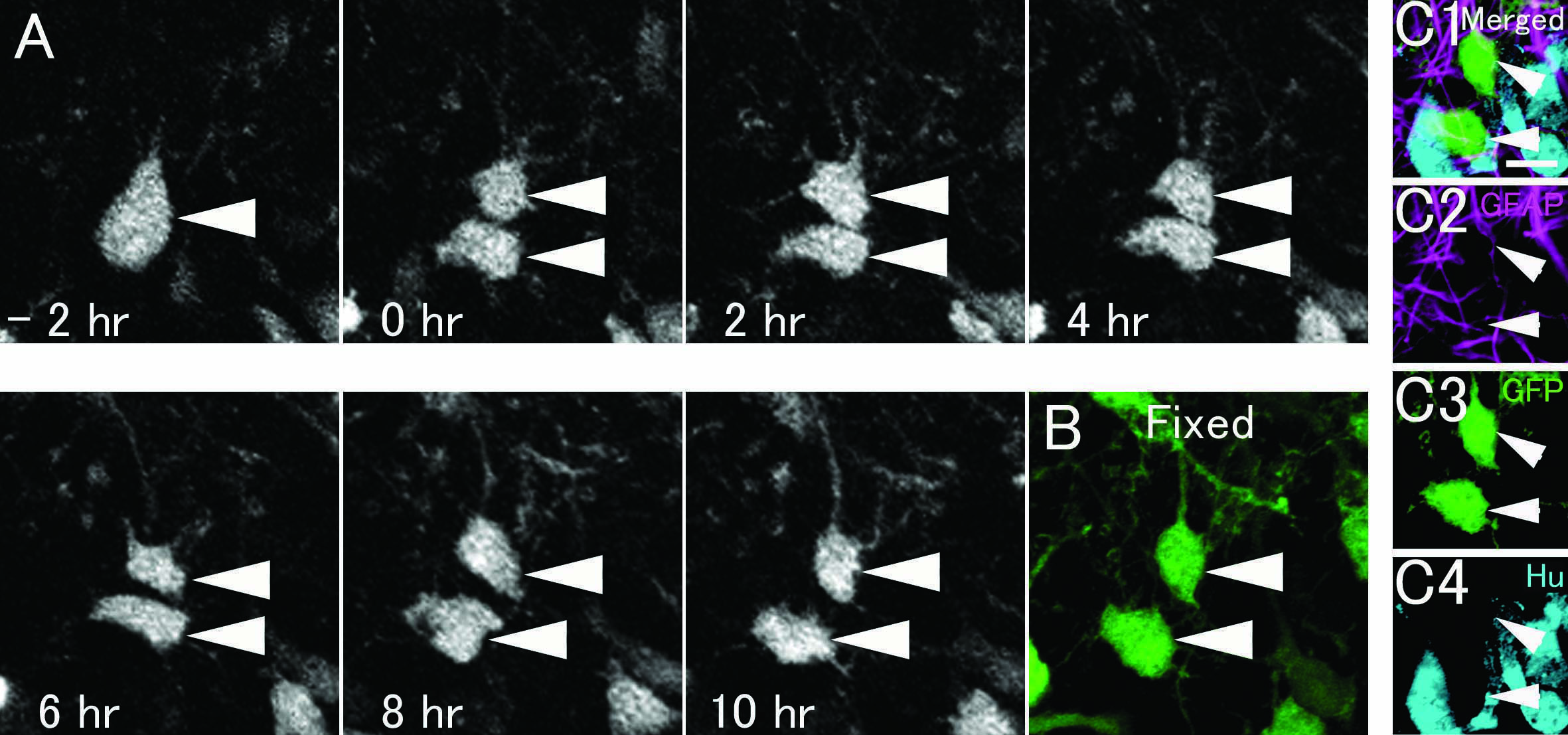

Supplement: Figure S3 — Time-lapse imaging of eGFP+ cells (A) and daughter cell fates at the end of culture (B, C). A: Full time-scale images of eGFP+ cells represented in Fig. 1C. B, C: Two eGFP+ daughter cells at the end of imaging. Both daughter cells expressed GFAP (magenta), but not Hu (blue). (TIF) [file pone.0025303.s003.tif]

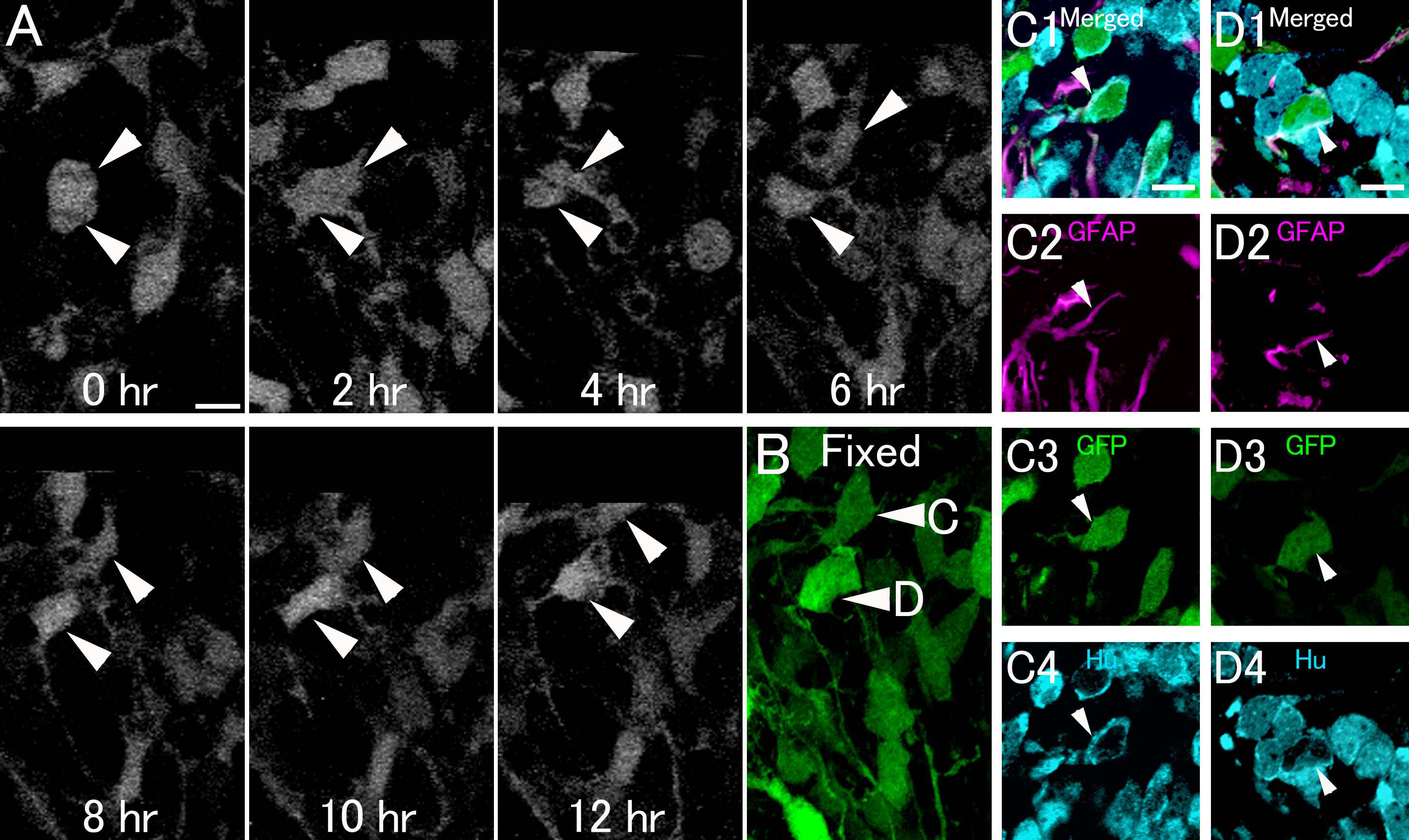

Supplement: Figure S4 — Time-lapse imaging of eGFP+ cells (A) and daughter cell fates at the end of culture (B, C). A: Full time-scale images of eGFP+ cells are represented in Fig. 1D. B, C, D: Two eGFP+ daughter cells at the end of the imaging. Both daughter cells expressed GFAP (magenta) and Hu (blue). (TIF) [file pone.0025303.s004.tif]

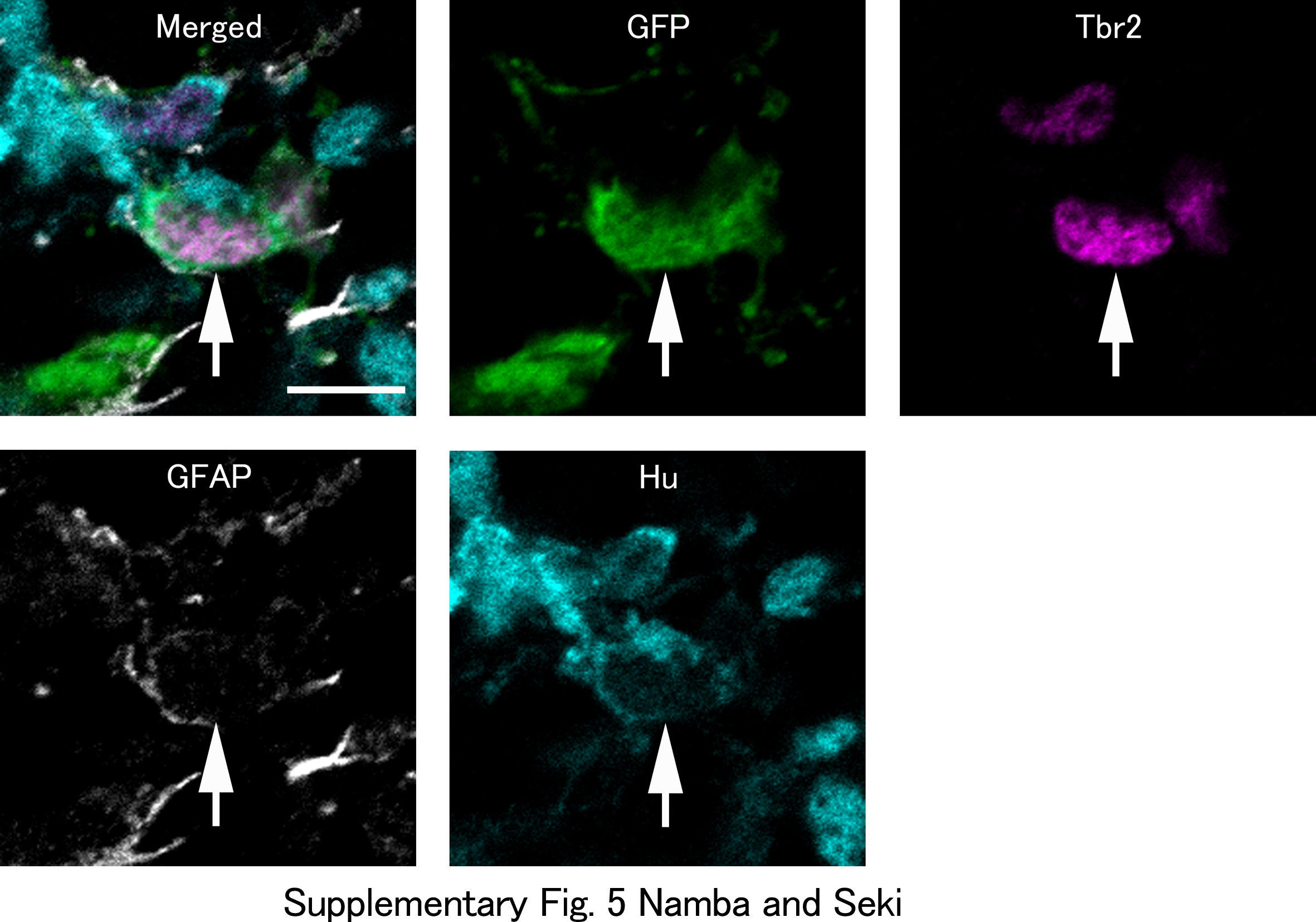

Supplement: Figure S5 — Phenotypic analysis of eGFP+ cells in the dentate gyrus at P5. The eGFP+ (green)/GFAP+ (white)/Hu+ (blue) cell indicated by arrow is also positive for Tbr2 (magenta). Scale bar, 5 µm. (TIF) [file pone.0025303.s005.tif]

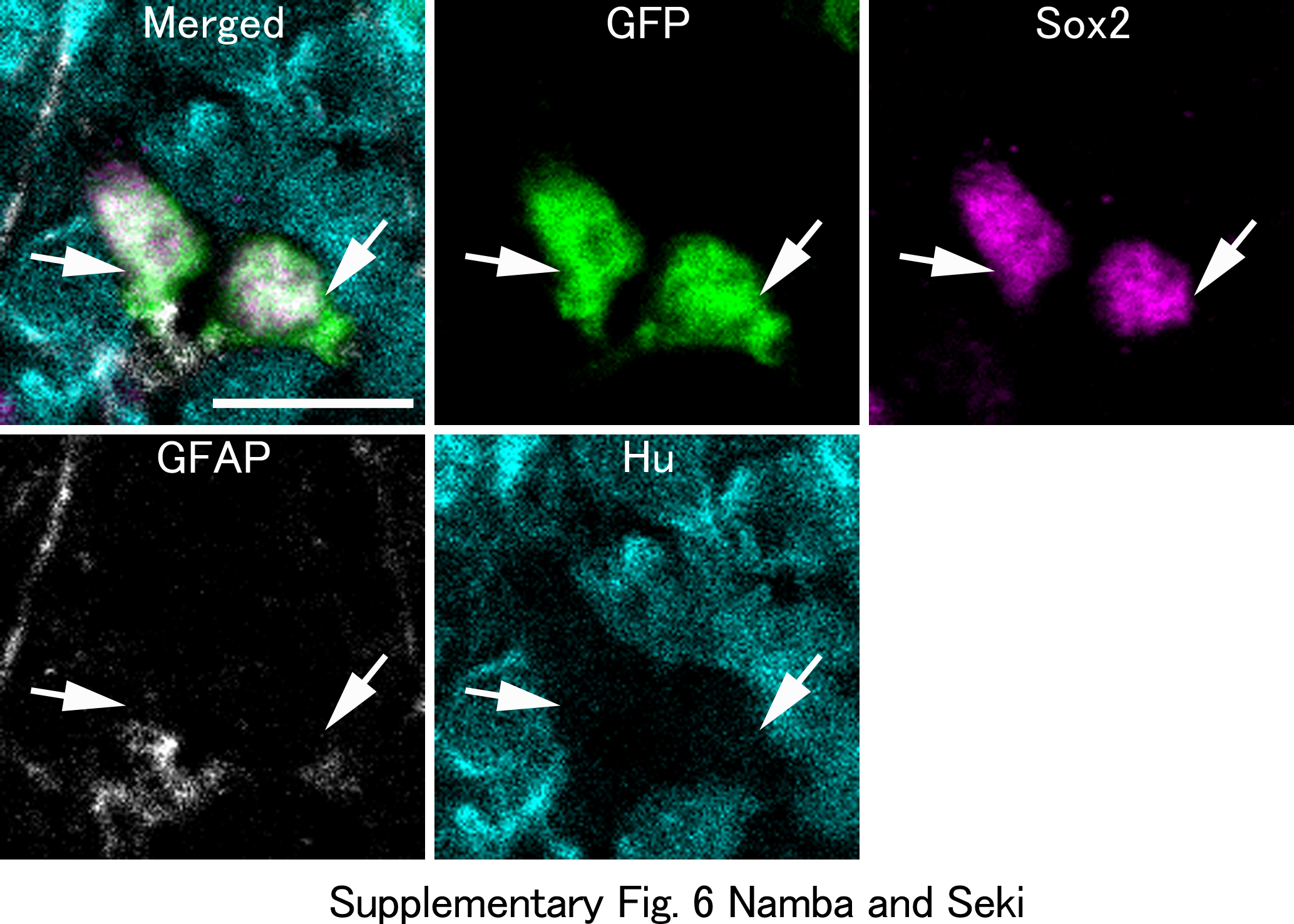

Supplement: Figure S6 — Phenotypic analysis of eGFP+ cells in the dentate gyrus at P5. The eGFP+ (green)/GFAP+ (white)/Hu− (blue) cells indicated by arrows are also positive for Sox2 (magenta). Scale bar, 10 µm. (TIF) [file pone.0025303.s006.tif]

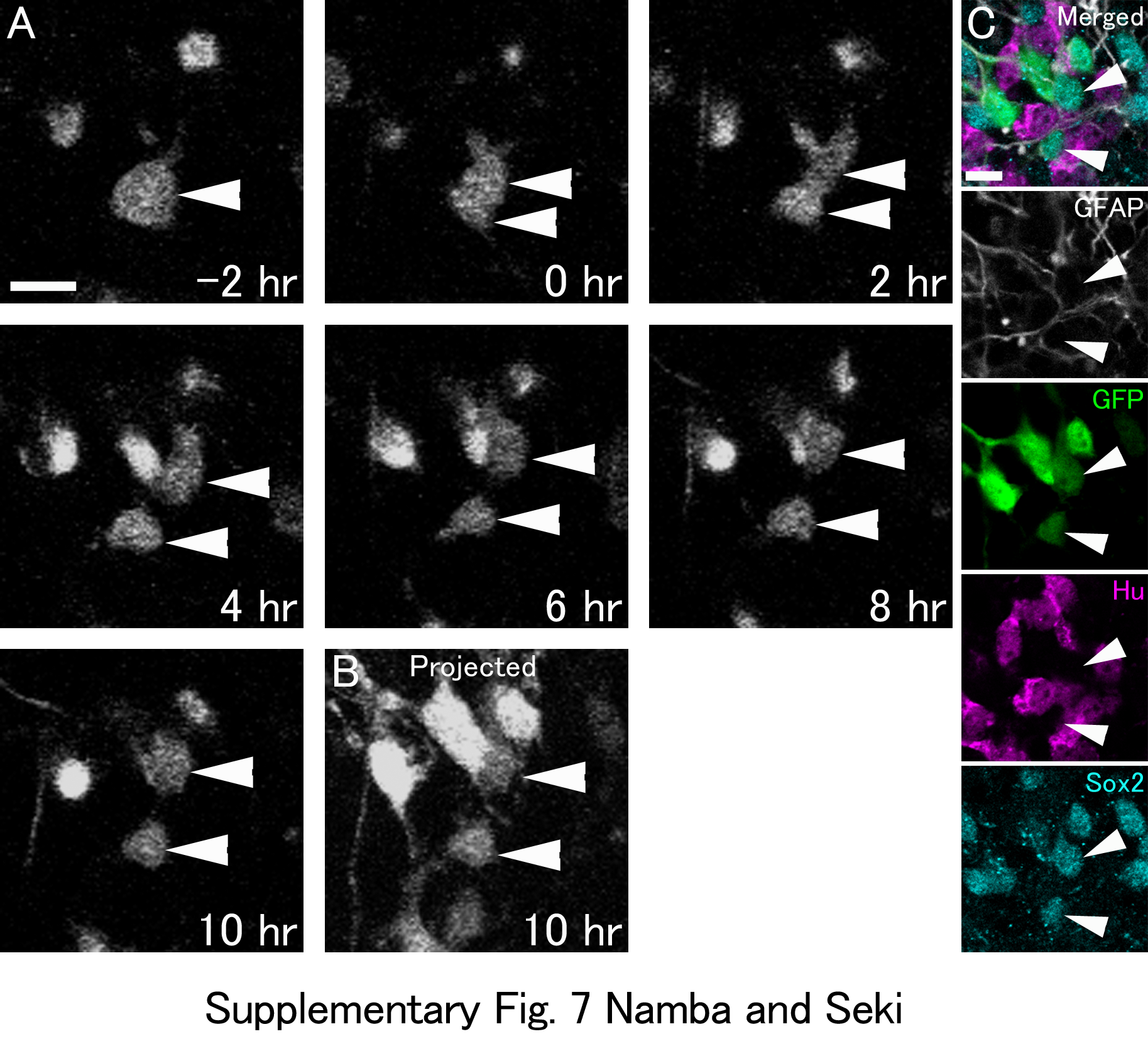

Supplement: Figure S7 — Symmetric division of eGFP+ cells to produce 2 GFAP+/Sox2+ cells. A, B: Time-lapse imaging of GFP+ cell division in a hippocampal slice from a P4 GFAP-eGFP Tg mouse. C: Both eGFP+ daughter cells (arrowheads) expressed an astrocytic cell marker (GFAP) and a progenitor cell marker (Sox2), suggesting the self-renewal of a progenitor cell. Scale bar, 5 µm. (TIF) [file pone.0025303.s007.tif]
